# Cancer environmental immunotherapy: starving tumor cell to death by targeting TGFB on immune cell

**DOI:** 10.1136/jitc-2021-002823

**Published:** 2021-06-18

**Authors:** Xing Huang, Gang Zhang, Tingbo Liang

**Affiliations:** 1 Department of Hepatobiliary and Pancreatic Surgery, the First Affiliated Hospital, Zhejiang University School of Medicine, Hangzhou, China; 2 Zhejiang Provincial Key Laboratory of Pancreatic Disease, the First Affiliated Hospital, Zhejiang University School of Medicine, Hangzhou, China

**Keywords:** immunotherapy, CD4-Positive T-Lymphocytes, tumor microenvironment, metabolic networks and pathways, immunomodulation

## Abstract

The blockage of intersectional communication between tumor and its metabolic and immune microenvironment is now considered a promising solution in treating cancer. Tumors have been identified as a special type of “wounds” that do not heal. Recent studies demonstrate that the lack of the transforming growth factor beta (TGFB) signaling pathway in CD4^+^ helper T cells induces the remodeling of the intratumoral vascular tissue, like healing “wounds” in damaged tissues caused by tumor overgrowth, which consequently prevents tumor cells from receiving the required nutrients in their microenvironment. TGFB blockade thereby promotes damaged tissue healing, causing tumor cell death as a result of starvation, ultimately obtaining an effective anticancer immunotherapy immune response. Here, we comment on the TGFB-mediated crosstalk between immune system and nutritional supply, highlighting cancer immunotherapeutic strategies targeting environmental immune-metabolism interplay. Cancer environmental immunotherapy targeting TGFB might therefore become one of the most promising treatment strategies for patients with cancer.

Hypoxic microenvironment in tumor stimulates surrounding angiogenesis to support nutrient requirements for its rapid growth. Already in the mid-1980s, the oncologist Harold Dvorak had revealed the similarity between tumor stroma formation and wound healing. According to his studies, tumor is essentially “non-healing wound”, a very forward-looking concept in that era.^1^ To date, it has been well established that wound healing follows an inflammatory reaction at the early stage of injury. Inflammatory cells exudate into the wound to remove necrotic tissues and kill the microorganisms that may cause infection. With cell proliferation and angiogenesis, the wound is gradually covered by freshly generated tissues in the later stage, and finally the inflammatory response decreases. However, as regard tumor, when the blood vessels are fully expanded into the damaged tissue caused by its overgrowth, it never enters the recovery phase of wound healing after initial inflammatory response. Instead, the tumor receives a continuous nutrient supply to support its growth through inducing nearby cell death and preventing the repair of surrounding tissues. Therefore, the finding of key factors or elements that prevent tumor tissue from accomplishing wound healing may represent a great possibility to develop a powerful drug or solution for cancer therapy.

Transforming growth factor beta (TGFB) plays an essential role in both wound healing and tumor development.^2^ As a critical regulatory factor of the inflammatory response, TGFB is essential for the maintenance of the immune homeostasis, the formation of immune tolerance, and the generation of local blood vessels. Its expression fluctuates within the inflammatory cycle during the wound healing process, whereas it persists in tumor. Interestingly, a recent study revealed that TGFB inhibition-caused tumor-killing effect is mediated by CD4^+^ helper T cells, rather than CD8^+^ effector T cells.^3^ Liu *et al* silenced the expression of TGFB receptors on CD4^+^ T cells and CD8^+^ T cells in mice with breast cancer and found that tumor growth is severely inhibited when CD4^+^ T cells, rather than CD8^+^ T cells, lack TGFB receptors. Intriguingly, once the intrinsic TGFB signaling pathway is blocked, CD4^+^ T cells were observed to promote the healing of tissues around the tumor. In this process, the growth of blood vessels is significantly reshaped and a dense “wall”-like protective layer is built around the blood vessel itself, which deprives tumor cells of receiving an adequate nutritional supply, consequently causing hypoxia-induced tumor cell death. Moreover, such tumor-killing effect depends on the stimulation of interleukin 4 production, instead of interferon γ. Considered complete inhibition of TGFB in body may cause serious side effects due to its multiple functions, Li *et al* further designed and constructed a bispecific therapeutic antibody called 4T-Trap,^4^ which simultaneously binds TGFB and helper T cells. In this way, it specifically inhibits the activation of the TGFB signaling pathway only on CD4^+^ T cells compared with traditional TGFB inhibitors targeting multiple cells. Indeed, it was observed that 4T-trap induced the reshaping of blood vessels around the breast tumor, as well as the hypoxia and death of tumor cells, thereby significantly inhibiting tumor growth in mice. Moreover, such specific approach significantly reduces the side effects caused by the treatment with mono-TGFB inhibitors. Overall, CD4^+^ helper T cells play an equally important role as CD8^+^ effector T cells in cancer immunity, and the inhibition of TGFB signaling pathway in CD4^+^ T cells can effectively trigger a complete wound healing response, causing tumor cell death through cutting off nutritional support. Based on these findings, the authors proposed a new type of therapy, “cancer environmental immunotherapy”, which seals the tumor microenvironment to starve tumor cells to death. It represents a significant complement to the treatment approaches in cancer therapy, promisingly in the combination with the existing immunotherapies in the future.

Accumulating evidence suggests that tumor can be divided into three phenotypes, such as “immune inflammation”, “immune rejection”, and “immune desert”, according to the “hot” or “cold” immune status in the environment.^5^ The immune checkpoint blockade, represented by targeting programmed cell death protein 1 (PD-1)/ PD-1 ligand 1 (PD-L1) axis, has become one of the most commonly used cancer therapy in clinic, and been approved as the first-line or second-line treatment for non-small-cell lung cancer, melanoma, urothelial cancer, renal cell carcinoma, head and neck cancer, Hodgkin lymphoma, and Merkel cell carcinoma.^6^ However, this approach mainly focuses on blocking the tumor’s inhibitory effect on the immune system to reactivate the immune system itself to identify and eliminate tumor cells, but not considering the huge influence of the microenvironment on both tumor and immune system. This is probably the crucial reason causing the lack of response or weak response of patients with cancer to this immune checkpoint therapy. In addition, there is no need to mention its well-recognized limitations in clinical practice. Therefore, immune checkpoint blockade-based combinational treatment is a potentially promising strategy to overcome therapeutic resistance.

As a matter of fact, Mariathasan *et al* had already reported that TGFB blockage significantly improves the efficacy of immune checkpoint inhibitors in multiple tumor types 2 years ago.^7^ The authors discovered that the activation of TGFB signaling pathway was negatively correlated with the immunotherapeutic response in metastatic urothelial cancer treated with the PD-L1 inhibitor atezolizumab. Further studies showed TGFB pathway activation in tumor microenvironmental fibroblasts reduces the infiltration of T cells into the tumor, thereby inhibiting the therapeutic efficacy of PD-L1 inhibition. The infiltration and distribution of T cells can be significantly enhanced by the combined inhibition of PD-L1 and TGFB, transforming the tumor from an immune rejection phenotype to an inflammatory one. Hence, cotargeting TGFB and immune checkpoints in cancer therapy synergistically results in tumor cell death. In another study, Tauriello and colleagues revealed a highly active TGFB signaling in colorectal tumor stroma and identified cancer-associated fibroblast cells as the main source of TGFB.^8^ Consistently, the combined administration of an anti-PD-L1 antibody and the TGFB type I receptor-specific inhibitor galunisertib in a metastatic mouse model induces lymphocyte infiltration in the tumor microenvironment and enhances the production of granzyme B^+^ and interferon γ^+^ effector T cells, eventually leading to metastatic tumor cell death and consequent survival of the mouse without any recurrence. Overall, these findings reveal that the inhibition of the TGFB signaling pathway can increase lymphocyte infiltration in tumor, thus bringing the tumor microenvironment to an inflammatory state, finally making it vulnerable to the PD-1/PD-L1 checkpoint blockade. At present, drug candidates are designed and developed by many pharmaceutical companies according to this strategy. For instance, MSB0011359C, a novel drug composed of the fusion of a monoclonal antibody targeting PD-L1 and a protein containing the extracellular domain of TGFB type II receptor, significantly improves the ability of immune cells to kill tumor cells, overcoming the resistance of tumor cells to PD-1/PD-L1 therapy. Moreover, as demonstrated by current preclinical and preliminary clinical studies, this drug reduces the side effects observed when two separated drugs are used for a combined medication inhibiting both the PD-1-PD-L1 axis and the TGFB signaling pathway.

Of note, although TGFB inhibition is well documented to attenuate tumor growth and metastasis in mice models and the field of TGFB-associated immuno-oncology is rapidly expanding, some preclinical findings showed adverse effects of TGFB-targeted therapies.^9^ For instance, TBR1 kinase inhibitor YL364947 and a pan-TGFB antibody increased mammary cancer metastasis and dissemination in mice, respectively, as a result of heterogeneity of tumor immune microenvironment within the same tumor or across distinct tumors. In addition, the potential influence on the homeostasis of immune system should also be taken into serious consideration, especially in allergy and autoimmunity-related diseases. Therefore, it is significant to identify biomarkers that predict response to TGFB blockage to distinguish the effectiveness of TGFB inhibition on tumor cell proliferation and dissemination. It is reported that immunotyping can indicate tumor immune microenvironment, thus predicting responses to immunotherapy.^10^ Accordingly, the construction of cancer immune subtypes might be favorable to selecting suitable patients for TGFB inhibition.

Altogether, TGFB acts as a “gatekeeper” of the tumor microenvironment, mediating the communication between the immune system and nutritional supply, thus becoming one of the most promising targets in inducing tumor cell death (figure 1). The development of TGFB-targeting drugs, as well as the design and optimization of combined treatment strategies, may greatly enhance the efficacy of cancer immunotherapy, thereby significantly improving the prognosis of patients, which warrants further exploration.

**Figure 1:**
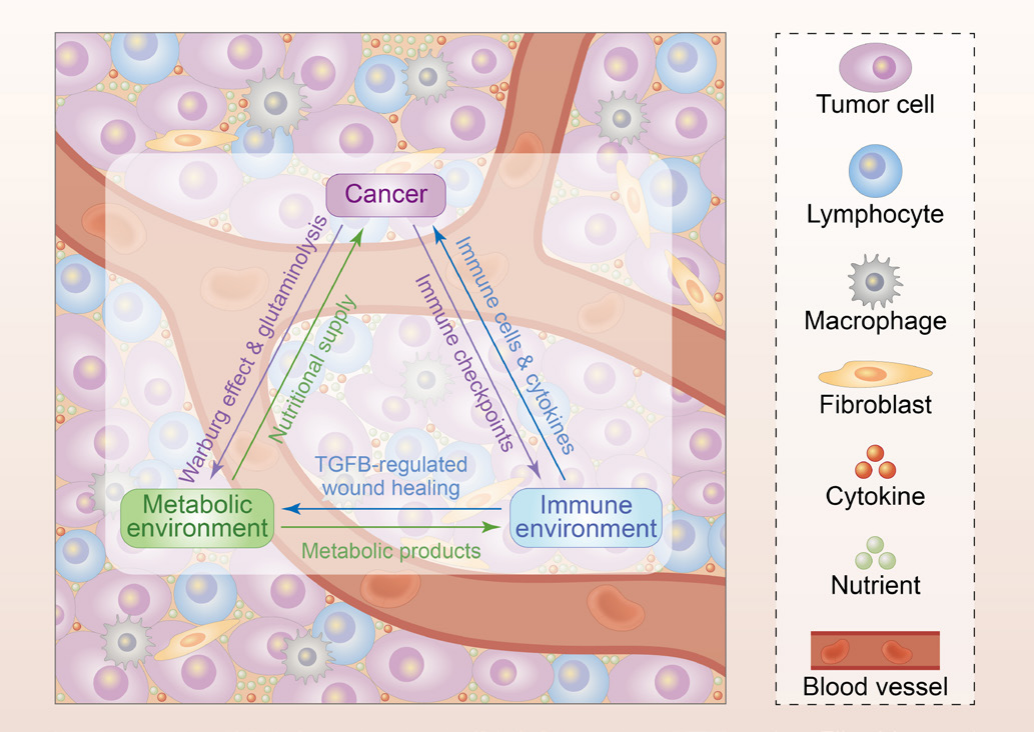
Immunological support of nutritional supply by TGFB in triangulation between cancer and its metabolic and immune microenvironment. Cancer metabolism leads to deprivation of nutrients and accumulation of specific metabolites in tumor microenvironment, which further causes the inactivation of immune effector cells and consequently cancer immune evasion. In reverse, immune checkpoints dominate the direct suppressive effects of cancer on immune system, while the activation of TGFB signaling pathway in CD4^+^ helper T cells inhibits tumor wound healing as well as wound healing-mediated blockade of nutritional supply in tumor microenvironment, thus immunologically supporting cancer metabolism. TGFB, transforming growth factor beta.
